# Associations between Rice, Noodle, and Bread Intake and Sleep Quality in Japanese Men and Women

**DOI:** 10.1371/journal.pone.0105198

**Published:** 2014-08-15

**Authors:** Satoko Yoneyama, Masaru Sakurai, Koshi Nakamura, Yuko Morikawa, Katsuyuki Miura, Motoko Nakashima, Katsushi Yoshita, Masao Ishizaki, Teruhiko Kido, Yuchi Naruse, Kazuhiro Nogawa, Yasushi Suwazono, Satoshi Sasaki, Hideaki Nakagawa

**Affiliations:** 1 Department of Epidemiology and Public Health, Kanazawa Medical University, Ishikawa, Japan; 2 Department of Health Science, Shiga University of Medical Science, Otsu, Japan; 3 Department of Community Health Nursing, School of Nursing, Kanazawa Medical University, Ishikawa, Japan; 4 Department of Food and Human Health Science Osaka City University, Graduate School of Human Life Science, Osaka, Japan; 5 Department of Social and Environmental Medicine, Kanazawa Medical University, Ishikawa, Japan; 6 School of Health Science, College of Medical, Pharmaceutical and Health Science, Kanazawa University, Kanazawa, Japan; 7 Department of Human Science and Fundamental Nursing, Toyama University School of Nursing, Toyama, Japan; 8 Department of Occupational and Environmental Medicine, Graduate School of Medicine, Chiba University, Chiba, Japan; 9 Department of Social and Preventive Epidemiology, the University of Tokyo, Tokyo, Japan; University of Louisville, United States of America

## Abstract

**Background:**

Previous studies have shown that a diet with a high-glycemic index is associated with good sleep quality. Therefore, we investigated the association of sleep quality with the intake of 3 common starchy foods with different glycemic indexes–rice, bread, and noodles–as well as the dietary glycemic index in a Japanese population.

**Methods:**

The participants were 1,848 men and women between 20 and 60 years of age. Rice, bread, and noodle consumption was evaluated using a self-administered diet history questionnaire. Sleep quality was evaluated by using the Japanese version of the Pittsburgh Sleep Quality Index, and a global score >5.5 was considered to indicate poor sleep.

**Results:**

Multivariate-adjusted odds ratios (95% confidence intervals) for poor sleep across the quintiles of rice consumption were 1.00 (reference), 0.68 (0.49–0.93), 0.61 (0.43–0.85), 0.59 (0.42–0.85), and 0.54 (0.37–0.81) (*p* for trend = 0.015); those for the quintiles of noodle consumption were 1.00 (reference), 1.25 (0.90–1.74), 1.05 (0.75–1.47), 1.31 (0.94–1.82), and 1.82 (1.31–2.51) (*p* for trend = 0.002). Bread intake was not associated with sleep quality. A higher dietary glycemic index was significantly associated with a lower risk of poor sleep (*p* for trend = 0.020).

**Conclusion:**

A high dietary glycemic index and high rice consumption are significantly associated with good sleep in Japanese men and women, whereas bread intake is not associated with sleep quality and noodle consumption is associated with poor sleep. The different associations of these starchy foods with sleep quality might be attributable to the different glycemic index of each food.

## Introduction

Sleep quality is known to be a function of sleep duration and latency [1]. Epidemiological studies have shown that short sleep duration is associated with increased mortality [2], [3], poor mood [2], [4], chronic health conditions (e.g., obesity and metabolic syndrome) [2], [5], cardiovascular disease and diabetes [2], [6], hypertension [2], [7], and poor self-related health and quality of life [2], [8]. Moreover, previous studies have shown that dietary factors affect sleep quality. A cross-sectional study of children younger than 2 years of age showed that the consumption of a meal with a high-glycemic index (GI) in the evening was associated with longer sleep duration [9]. In addition a clinical trial showed that sleep onset latency is reduced by approximately 10 minutes after the consumption of a carbohydrate-rich evening meal with a high-GI compared with an evening meal with a low-GI [10]. These data suggest that sleep quality is influenced by the carbohydrate-based GI of the meals.

The dietary GI of the general Japanese population is approximately 70 [11]–[13], this is considerably higher than the dietary GI in predominantly Western populations (i.e., European, Australian, and North American), which ranges from 48 to 60 [14]–[16]. This difference may be due to disparities in the average intakes of different foods that contribute to the GI. Rice is a common starchy food in the Japanese diet, as approximately 70% of the cereals consumed are rice [17], and rice accounts for 59% of the dietary GI [11]–[13]. However, no studies have evaluated the associations of rice or other common staple foods such as bread and noodles with sleep quality in a Japanese population.

This study investigated the associations between sleep quality and intake of carbohydrate-based staple foods (i.e., rice, bread, and noodles) as well as the dietary GI and glycemic load (GL) in a Japanese population.

## Methods

### Participants

The study population comprised 7,306 employees of a factory that produces zippers and aluminum sashes in Toyama Prefecture, Japan. The Industrial Health and Safety Law in Japan requires that employers offer annual health examinations to all of their employees. The present study included data on 2,255 white-collar daytime workers between 20 and 60 years of age. White-collar workers were studied because many blue-collar workers are involved in shift work, making it difficult to evaluate sleep quality.

A questionnaire about diet was completed by 1,977 (88%) of the white-collar daytime workers in 2003, and a questionnaire about sleep was completed by 2003 (94%) of the worker in 2004. In total, 1,858 (82%) of the workers provided complete data on both questionnaires. Ten participants with extremely low or high energy intake (i.e., <500 or >4,000 kcal/day) were excluded from the study. Thus, data on 1,848 white-collar daytime workers (1,164 men and 684 women) were included in the analysis.

### Data collection

The annual health examination included a medical history, physical examination, and anthropometric measurements. Body mass index was calculated as weight divided by height squared (kg/m^2^). A questionnaire was used to identify health-related behaviors including smoking status (i.e., current, previous, or never), and habitual exercise; habitual exercise was assessed as hours per week spent on leisure time physical activities and was expressed as metabolic equivalent hours per week (MET-h/week).

### Dietary assessment and calculation of the dietary GI and GL

A self-administered diet history questionnaire (DHQ) was used to assess dietary habits during the preceding month. [18], [19]. The DHQ was developed to estimate the respondents’ dietary intake of macronutrients and micronutrients for use in epidemiological studies in Japan. Detailed descriptions of the methods used to calculate dietary intakes and the validity of the DHQ have been reported previously [18], [19]. Estimates of dietary intake for 147 food and beverage items, energy, protein, fat, total carbohydrate, and alcohol in 2003 were calculated using an ad hoc computer algorithm developed for the DHQ that was based on the Standard Tables of Food Composition in Japan [20]. The DHQ evaluates the consumption of 19 staple foods (i.e., rice, noodles, and other wheat foods). The intake frequency of each staple food for breakfast, lunch, dinner, and snack/midnight snack in 1 week was evaluated. For rice, the type of rice (i.e., white rice, white rice mixed with barley, white rice with germ, 50% polished rice, 70% polished rice, or brown rice) and serving size (i.e., number and size; cups for children, women, and men, and small and large bowls were defined as 110, 140, 170, 220, and 250 g, respectively) were evaluated. Similarly, the types of bread and noodles were evaluated. Bread was classified as white bread, buttered bread, cake bread, bread containing cream and sweet bean paste, pizza, *okonomiyaki* (Japanese “pizza,” which contains shredded cabbage and dough cooked in a frying pan), or Japanese-style pancakes (small pancakes containing flour, sugar, and egg, cooked in a frying pan). Noodles were classified as Japanese noodles (i.e., buckwheat and Japanese white noodles), instant noodles, Chinese noodles, or pasta [12]. The DHQ also includes the frequencies of skipping breakfast, lunch, dinner per week. Of the 147 food and beverage items included in the DHQ, 6 (4.1%) are alcoholic beverages, 8 (5.4%) contain no available carbohydrates, and 63 (42.9%) contain less than 3.5 g available carbohydrate per serving. Therefore, the dietary GI and GL were calculated on the basis of the remaining 70 items [11], [21]. The GI databases used were an international table of GI [22], a report on the GI values of Japanese foods [23], a report on GI values published after the publication of the international GI tables [24], and an online database provided by the Sydney University Glycemic Index Research Service [25]. The GIs of all foods in the DHQ have been published elsewhere [11]. Although there are concerns regarding the utility of the GI for mixed meals (i.e., overall diet) [26], [27], many studies have shown that the GI of mixed meals can be predicted on the basis of the GI value of each of the component foods [28]. We calculated the dietary GI as the sum of the percentage contribution of each food multiplied by their respective GI values. Dietary GL was calculated by multiplying the dietary GI by the total daily carbohydrate intake and dividing by 100. We used energy-adjusted values calculated using the density method (per 1,000 kcal) for GL [21]. The reproducibility and relative validity of the dietary GI and GL assessed using the DHQ have been reported elsewhere [21].

### Sleep assessment

Sleep quality in the previous month was assessed by using the Japanese version of the Pittsburgh Sleep Quality Index questionnaire (PSQI-J) [29], which was developed from the original questionnaire, the PSQI [1]. In brief, the PSQI-J is a standardized self-administered questionnaire for assessing sleep quality that includes the following 7 components: subjective sleep quality, sleep latency, sleep duration, habitual sleep efficiency, sleep disturbances, use of sleep medication, and daytime dysfunction. Each component is weighted equally on a scale of 0 to 3, and the scores for each component are then summed to yield a PSQI-J global score ranging from 0 to 21, higher scores indicate poorer sleep quality. Participants completed the PSQI-J at home in 5–10 minutes, and the responses were then reviewed by a well-trained nurse.

### Statistical analysis

When the PSQI-J component scores of the male and female participants were compared, sleep duration was significantly shorter in men than in women (*p* = 0.011); however, there were no significant differences in the other component scores including the PSQI-J global score between sexes. Therefore, all analyses were performed in the whole population, (i.e., not stratified by sex). In this study, we used energy density values for macronutrients and alcohol (% of energy [% energy]) and for food intakes (weight per 1000 kcal [g/1000 kcal]).

“Poor sleep,” was defined as a PSQI-J global score >5.5 [29]. In a previous study, using a cut-off of 5.5 for the PSQI-J global score provided estimates with a sensitivity and specificity of 85.7% and 86.6% for primary insomnia, 80.0% and 86.6% for major depression, and 83.3% and 86.6% for schizophrenia, respectively [29]. Rice, bread, and noodle intake as well as the GI and GL were categorized into quintiles. Analysis of covariance was used to evaluate the means of each PSQI-J component score adjusted for age, sex and total energy intake. The prevalence of poor sleep in each quintile was compared using the *χ* ^2^ test. Odds ratios (ORs) with 95% confidence intervals (95% CIs) for poor sleep were calculated using multiple logistic regression analyses. ORs were first adjusted for age (years; continuous) and sex (age- and sex-adjusted model) and then for body mass index (kg/m^2^; continuous), smoking status (i.e., current, previous, or never; dummy variable), habitual exercise (MET-h/week; continuous), alcohol consumption (percentage of energy; continuous), frequency of breakfast consumption per week (i.e., 0–3, 4–6, or 7 days/week; dummy variable), rice intake (for the multivariate analyses of bread and noodle intake; continuous), bread intake (for the multivariate analyses of rice and noodle intake; continuous), and noodle intake (for the multivariate analyses of rice and bread intake; continuous). The *p*-values for linear trends were calculated by using the median value of each quintile. Furthermore, each food, dietary GI, and dietary GL was included in the logistic regression analyses as continuous variables, and the ORs for an increment of 1 standard deviation in these variables were calculated. Statistical analyses were performed with Statistical Analysis System version 9.3 (SAS Institute Inc., Cary, NC, USA). The level of significance was set at p<0.05.

### Ethical considerations

Written informed consent was not obtained from the participants. The design of the present study was approved by the occupational safety and health committee of the subject company, which consisted of employee representatives. Employees were informed of the study design and of the right to refuse to participate in the study in the study documents. Participants who answered the questionnaire were regarded as having consented to the survey. Linkable anonymized data were provided by the company to ensure that individuals would not be identifiable by the researchers. This study was approved by the Institutional Review Committee of Kanazawa Medical University for Ethical Issues.

## Results

Table 1 shows the characteristics of the 1,848 study participants stratified by rice, bread, and noodle consumption quintiles as well as dietary GI and GL quintiles. Higher rice intake was significantly associated with older age (*p* = 0.001), higher carbohydrate intake (*p*<0.001), higher dietary GI (*p*<0.001), higher GL (*p*<0.001), lower bread intake (*p*<0.001), lower noodle intake (*p*<0.001), lower frequency of breakfast consumption (*p*<0.001), and a lower probability of being a current smoker (*p* = 0.026). Body mass index, alcohol consumption, and habitual exercise were not significantly associated with rice intake. Higher bread intake was significantly associated with a higher frequency of breakfast consumption (*p*<0.001), female sex (*p*<0.001), younger age (*p* = 0.045), lower alcohol consumption (*p*<0.001), lower rice intake (*p*<0.001), lower dietary GI (*p*<0.001), and lower dietary GL (*p*<0.001). Habitual exercise and smoking status were not significantly associated with bread intake. Higher noodle intake was significantly associated with higher carbohydrate intake (*p*<0.001), higher bread intake (*p* = 0.008), higher alcohol consumption (*p* = 0.012), lower rice intake (*p*<0.001), and lower dietary GI (*p*<0.001). Sex, smoking status, and frequency of breakfast consumption were not significantly associated with noodle intake. Increasing GI quintiles were significantly associated with older age (*p*<0.001), alcohol consumption (*p*<0.001), higher carbohydrate intake (*p*<0.001), higher rice intake (*p*<0.001), lower bread intake (*p*<0.001), lower noodle intake (*p*<0.001), and lower frequency of breakfast consumption (*p*<0.001). Habitual exercise and smoking status were not significantly associated with the dietary GI.

**Table 1:** Characteristics of study participants by quintiles of dietary rice, bread, and noodle intake as well as dietary glycemic index and glycemic load (*N* = 1,848).

	Q1 (lowest)			Q2			Q3 (middle)			Q4			Q5 (highest)			p value^a^
Rice (range, g/1,000 kcal)	<132.7			132.7–168.4			168.5–202.2			202.3–249.1			≥249.2			
Male (%)	48.0			54.5			61.7			74.1			76.7			<0.001
Age (years)	37.6	±	10.1	39.2	±	9.5	39.9	±	9.8	39.9	±	9.9	41.7	±	10.2	0.001
Body mass index (kg/m^2^)	22.4	±	3.1	22.5	±	3.0	23.1	±	3.2	22.9	±	3.2	23.0	±	3.0	0.060
Habitual exercise level (METs/week)	1.0	±	12.1	1.1	±	8.7	0.6	±	4.0	0.8	±	9.5	1.2	±	8.5	0.823
Current smokers (%)	28.0			26.6			30.4			32.8			37.5			0.026
Skip breakfast (%)	64.5			42.1			35.9			18.3			11.7			<0.001
Total energy intake (kcal/day)	2012	±	582	2034	±	491	1942	±	452	1870	±	410	1731	±	414	<0.001
Protein intake (%kcal)	17.9	±	2.9	17.8	±	2.6	17.2	±	2.5	16.5	±	2.4	15.5	±	2.4	<0.001
Fat intake (%kcal)	31.8	±	6.4	29.7	±	5.4	26.9	±	5.4	23.8	±	5.0	19.8	±	4.9	<0.001
Carbohydrate intake (%kcal)	52.0	±	7.1	54.5	±	5.6	57.3	±	5.4	60.3	±	5.6	65.3	±	5.9	<0.001
Alcohol consumption (%kcal)	5.0	±	7.5	4.4	±	6.4	4.5	±	6.6	4.7	±	5.8	4.1	±	6.1	0.603
Bread intake (g/1,000 kcal)	44.9	±	32.2	35.4	±	24.5	30.2	±	24.0	20.9	±	21.8	12.9	±	18.9	<0.001
Noodle intake (g/1,000 kcal)	47.9	±	38.1	37.7	±	30.4	34.4	±	29.6	37.3	±	35.7	27.0	±	29.0	<0.001
Dietary GI	63.8	±	4.1	66.6	±	2.7	68.1	±	2.9	69.7	±	2.5	71.6	±	3.0	<0.001
Dietary GL (/1,000 kcal)	141	±	46	157	±	40	163	±	42	171	±	39	179	±	44	<0.001
Bread (range, g/1,000 kcal)	0			0.1–14.8			14.9–30.1			30.2–47.2			≥47.3			
Male (%)	73.9			66.1			58.0			58.0			58.5			<0.001
Age (years)	41.1	±	10.0	39.9	±	9.8	39.7	±	9.9	39.5	±	10.2	38.9	±	9.8	0.045
Body mass index (kg/m^2^)	23.1	±	3.0	23.0	±	3.3	22.8	±	3.2	22.6	±	3.2	22.5	±	2.9	0.082
Habitual exercise level (METs/week)	1.3	±	10.7	0.5	±	2.4	1.3	±	10.5	0.7	±	5.1	0.9	±	10.3	0.610
Current smokers (%)	40.7			29.5			29.2			26.0			29.2			0.284
Skip breakfast (%)	17.3			10.7			20.6			50.9			72.4			<0.001
Total energy intake (kcal/day)	1811	±	445	2031	±	508	1995	±	508	1927	±	405	1803	±	472	<0.001
Protein intake (%kcal)	16.3	±	3.0	17.0	±	2.7	17.2	±	2.7	17.3	±	2.4	16.8	±	2.3	<0.001
Fat intake (%kcal)	22.5	±	7.1	25.7	±	6.5	27.3	±	6.5	27.8	±	6.2	26.6	±	6.2	<0.001
Carbohydrate intake (%kcal)	59.0	±	8.6	57.8	±	7.5	58.2	±	7.0	57.4	±	6.5	59.2	±	6.9	0.003
Alcohol consumption (%kcal)	7.43	±	8.36	5.15	±	6.80	3.12	±	4.61	3.40	±	4.86	3.28	±	5.38	<0.001
Rice intake (g/1,000 kcal)	239.1	±	71.4	206.3	±	62.9	185.6	±	61.3	165.1	±	52.7	154.8	±	59.5	<0.001
Noodle intake (g/1,000 kcal)	35.3	±	39.6	35.6	±	27.5	37.9	±	29.8	36.1	±	32.1	34.7	±	33.0	0.733
Dietary GI	70.1	±	4.2	68.9	±	3.6	67.9	±	3.6	67.3	±	3.6	67.2	±	3.6	<0.001
Dietary GL (/1,000 kcal)	160	±	44	172	±	45	170	±	46	161	±	39	157	±	42	<0.001
Noodles (range, g/1,000 kcal)	<7.4			7.4–23.3			23.4–36.5			36.6–57.4			≥57.5			
Male (%)	66.0			56.5			62.8			63.3			66.4			0.330
Age (years)	40.9	±	10.2	39.9	±	10.0	40.2	±	9.5	38.6	±	10.0	39.7	±	9.9	0.037
Body mass index (kg/m^2^)	22.9	±	3.1	22.9	±	3.1	22.6	±	2.9	22.7	±	3.1	23.0	±	3.4	0.498
Habitual exercise level (METs/week)	0.6	±	7.2	1.2	±	10.9	0.8	±	5.4	1.2	±	8.7	0.8	±	9.8	0.864
Current smokers (%)	26.2			27.3			32.2			33.0			27.9			0.801
Skip breakfast (%)	39.5			37.8			28.8			33.4			32.8			0.584
Total energy intake (kcal/day)	1833	±	455	1945	±	470	1997	±	421	1917	±	509	1861	±	509	<0.001
Protein intake (%kcal)	16.6	±	2.9	17.3	±	2.6	17.0	±	2.6	16.9	±	2.8	16.8	±	2.4	0.006
Fat intake (%kcal)	25.5	±	6.9	27.4	±	6.5	26.9	±	6.6	26.0	±	6.7	23.9	±	6.5	<0.001
Carbohydrate intake (%kcal)	58.8	±	7.6	57.8	±	6.9	57.1	±	6.8	58.3	±	7.3	59.8	±	7.9	<0.001
Alcohol consumption (%kcal)	4.72	±	6.90	3.48	±	5.39	4.86	±	6.77	4.52	±	6.38	4.95	±	6.40	0.012
Rice intake (g/1,000 kcal)	220.1	±	72.8	188.8	±	66.3	185.1	±	64.0	184.3	±	64.5	174.7	±	69.0	<0.001
Bread intake (g/1,000 kcal)	25.9	±	28.2	31.9	±	27.9	25.6	±	22.1	27.0	±	25.0	27.4	±	26.7	0.008
Dietary GI	70.5	±	4.0	68.9	±	3.5	68.5	±	3.4	67.6	±	3.4	66.0	±	3.7	<0.001
Dietary GL (/1,000 kcal)	163	±	41	166	±	43	168	±	41	163	±	44	159	±	50	0.094
Dietary GI (range)	<65.3			65.3–67.7			67.8–69.5			69.6–71.6			≥71.7			
Male (%)	52.4			53.5			62.3			69.2			77.5			0.011
Age (years)	38.7	±	10.3	40.2	±	9.6	39.1	±	9.6	39.6	±	10.0	41.6	±	9.9	<0.001
Body mass index (kg/m^2^)	22.6	±	3.3	22.7	±	3.2	22.7	±	2.9	23.0	±	3.3	23.1	±	2.8	0.172
Habitual exercise level (METs/week)	1.3	±	12.7	0.4	±	1.9	1.1	±	10.6	0.6	±	3.8	1.2	±	8.9	0.466
Current smokers (%)	27.5			24.9			33.7			31.5			38.3			0.605
Skip breakfast (%)	50.8			39.2			35.5			28.4			18.4			<0.001
Total energy intake (kcal/day)	2017	±	560	1977	±	469	1896	±	449	1858	±	426	1804	±	439	<0.001
Protein intake (%kcal)	17.7	±	2.7	17.9	±	2.5	16.8	±	2.3	16.5	±	2.5	15.7	±	2.6	<0.001
Fat intake (%kcal)	28.7	±	6.6	27.9	±	6.2	26.4	±	6.4	24.5	±	6.2	22.3	±	6.4	<0.001
Carbohydrate intake (%kcal)	56.0	±	7.4	56.9	±	6.9	58.8	±	7.1	59.0	±	6.9	61.1	±	7.4	<0.001
Alcohol consumption (%kcal)	4.1	±	6.3	3.6	±	5.3	3.6	±	5.2	5.5	±	7.5	5.7	±	7.1	<0.001
Rice intake (g/1,000 kcal)	128.9	±	55.5	160.4	±	44.0	187.3	±	50.2	214.9	±	46.8	261.7	±	61.5	<0.001
Bread intake (g/1,000 kcal)	35.5	±	29.2	32.6	±	24.8	30.6	±	26.0	24.5	±	24.1	14.6	±	20.6	<0.001
Noodle intake (g/1,000 kcal)	53.9	±	40.2	46.5	±	34.0	34.5	±	28.8	27.3	±	23.7	17.4	±	19.3	<0.001
Dietary GL (range,/1,000 kcal)	<128			128–149			150–169			170–194			≥195			
Male (%)	47.8			48.1			64.8			70.3			84.0			<0.001
Age (years)	37.7	±	9.7	39.4	±	9.7	40.4	±	9.6	41.3	±	10.1	40.4	±	10.2	<0.001
Body mass index (kg/m^2^)	22.0	±	3.1	22.5	±	3.1	23.1	±	3.3	23.1	±	3.0	23.4	±	2.9	<0.001
Habitual exercise level (METs/week)	0.4	±	2.6	0.8	±	7.7	0.3	±	1.1	1.3	±	12.4	2.0	±	12.3	<0.001
Current smokers (%)	33.7			27.4			27.7			33.9			33.2			0.301
Skip breakfast (%)	58.9			36.8			31.7			24.1			20.9			<0.001
Total energy intake (kcal/day)	1472	±	305	1695	±	272	1870	±	302.9	2052	±	331	2465	±	450	<0.001
Protein intake (%kcal)	17.5	±	3.0	17.2	±	2.6	17.1	±	2.6	16.8	±	2.5	15.9	±	2.4	<0.001
Fat intake (%kcal)	28.2	±	7.1	26.6	±	6.4	25.9	±	6.6	25.1	±	6.6	24.0	±	6.4	<0.001
Carbohydrate intake (%kcal)	53.8	±	7.4	57.6	±	6.5	58.5	±	6.8	59.8	±	6.8	62.1	±	6.7	<0.001
Alcohol consumption (%kcal)	6.5	±	8.8	4.3	±	5.7	4.2	±	6.1	4.0	±	5.2	3.5	±	5.0	<0.001
Rice intake (g/1,000 kcal)	154.3	±	64.4	189.4	±	64.3	197.1	±	66.5	203.4	±	65.0	208.9	±	71.6	<0.001
Bread intake (g/1,000 kcal)	30.7	±	28.5	27.1	±	24.7	29.8	±	28.1	24.4	±	23.5	25.9	±	25.2	0.004
Noodle intake (g/1,000 kcal)	41.8	±	35.7	36.3	±	30.9	31.1	±	30.9	34.3	±	31.1	36.1	±	34.4	<0.001

GI: glycemic index, GL: glycemic load, METs/week: metabolic equivalent hours per week; Q: quintile.

a The *χ* ^2^ test was used to analyze categorical variables, and linear regression analysis was used to calculate *p*-values for trends for continuous variables.

**Figure 1:**
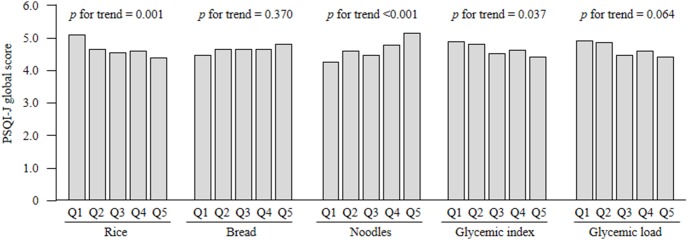
PSQI-J global scores for quintiles of starchy food intake, dietary glycemic index, and glycemic load. Mean PSQI-J global scores were adjusted for age, sex, and total energy intake (kcal/day, continuous), by using the analysis of covariance model. PSQI-J: Japanese version of the Pittsburgh Quality Index; Q: quintile.

Figure 1 shows the mean PSQI-J global score according to rice, bread, and noodle consumption quintile as well as dietary GI and GL quintiles adjusted for age, sex and total energy intake. Higher rice intake and higher GI were significantly associated with a lower PSQI-J global score (*p* = 0.001 and *p = 0*.037, respectively), and higher noodle intake was significantly associated with a higher PSQI-J global score (*p*<0.001). Table 2 shows sleep duration and PSQI-J component scores according to rice, bread, and noodle consumption quintile as well as dietary GI and GL quintile. Higher rice intake was significantly associated with lower scores for poor sleep duration (*p* = 0.003) but was not associated with any other component of sleep quality. Higher noodle intake was significantly associated with a higher frequency of sleep disturbance (*p*<0.001), higher levels of daytime dysfunction (*p* = 0.005), increased use of sleep medication (*p* = 0.008), poorer subjective sleep quality (*p* = 0.021), and longer sleep latency (*p* = 0.049). A higher dietary GI was significantly associated with lower scores for poor sleep duration (*p* = 0.013) but not with other PSQI-J components. These associations remained even after adjusting for age, sex, and total energy intake (data not tabulated).

**Table 2:** PSQI-J component scores by quintiles of rice, bread, and noodle intake as well as dietary glycemic index and glycemic load.

	Q1 (lowest)			Q2			Q3 (middle)			Q4			Q5 (highest)			P value^a^
Rice																
Sleep duration (hours)	6.2	±	0.9	6.3	±	0.8	6.4	±	0.8	6.4	±	0.9	6.4	±	0.8	0.008
PSQI-J																
Subjective sleep quality	1.26	±	0.69	1.16	±	0.64	1.18	±	0.68	1.15	±	0.64	1.08	±	0.63	0.063
Sleep latency	0.72	±	0.77	0.70	±	0.74	0.70	±	0.77	0.67	±	0.74	0.67	±	0.75	0.630
Sleep duration	1.04	±	0.84	0.91	±	0.74	0.81	±	0.70	0.84	±	0.75	0.79	±	0.73	0.003
Habitual sleep efficiency	0.13	±	0.50	0.06	±	0.25	0.07	±	0.31	0.08	±	0.34	0.09	±	0.40	0.787
Sleep disturbances	0.93	±	0.51	0.94	±	0.51	0.90	±	0.50	0.89	±	0.49	0.86	±	0.51	0.290
Use of sleep medication	0.08	±	0.40	0.04	±	0.26	0.05	±	0.33	0.06	±	0.36	0.03	±	0.29	0.773
Daytime dysfunction	0.99	±	0.73	0.91	±	0.67	0.87	±	0.74	0.96	±	0.72	0.86	±	0.71	0.211
Bread																
Sleep duration (hours)	6.4	±	0.8	6.4	±	0.8	6.3	±	0.8	6.3	±	0.8	6.2	±	0.8	0.007
PSQI-J																
Subjective sleep quality	1.13	±	0.69	1.17	±	0.60	1.14	±	0.64	1.17	±	0.65	1.22	±	0.69	0.407
Sleep latency	0.71	±	0.76	0.71	±	0.75	0.64	±	0.73	0.69	±	0.73	0.72	±	0.78	0.570
Sleep duration	0.77	±	0.75	0.83	±	0.74	0.90	±	0.74	0.92	±	0.78	0.97	±	0.78	0.003
Habitual sleep efficiency	0.07	±	0.30	0.10	±	0.39	0.10	±	0.37	0.09	±	0.41	0.08	±	0.37	0.706
Sleep disturbances	0.88	±	0.50	0.94	±	0.52	0.93	±	0.50	0.89	±	0.50	0.90	±	0.51	0.383
Use of sleep medication	0.05	±	0.33	0.05	±	0.32	0.05	±	0.33	0.04	±	0.29	0.06	±	0.38	0.905
Daytime dysfunction	0.89	±	0.72	0.94	±	0.71	0.97	±	0.73	0.91	±	0.71	0.89	±	0.69	0.491
Noodles																
Sleep duration (hours)	6.38	±	0.83	6.32	±	0.81	6.37	±	0.80	6.30	±	0.86	6.27	±	0.82	0.275
PSQI-J																
Subjective sleep quality	1.11	±	0.66	1.14	±	0.66	1.13	±	0.63	1.22	±	0.68	1.23	±	0.65	0.021
Sleep latency	0.64	±	0.72	0.65	±	0.70	0.67	±	0.75	0.72	±	0.79	0.79	±	0.78	0.049
Sleep duration	0.82	±	0.75	0.91	±	0.76	0.82	±	0.73	0.90	±	0.77	0.94	±	0.78	0.114
Habitual sleep efficiency	0.05	±	0.28	0.07	±	0.37	0.10	±	0.39	0.09	±	0.39	0.12	±	0.40	0.069
Sleep disturbances	0.81	±	0.52	0.94	±	0.51	0.87	±	0.50	0.95	±	0.49	0.96	±	0.50	<0.001
Use of sleep medication	0.02	±	0.23	0.03	±	0.27	0.03	±	0.28	0.06	±	0.36	0.10	±	0.46	0.008
Daytime dysfunction	0.83	±	0.72	0.91	±	0.70	0.92	±	0.72	0.90	±	0.71	1.03	±	0.70	0.005
Dietary GI																
Sleep duration (hours)	6.3	±	0.8	6.3	±	0.8	6.3	±	0.8	6.4	±	0.8	6.4	±	0.8	0.025
PSQI-J																
Subjective sleep quality	1.21	±	0.68	1.17	±	0.62	1.16	±	0.65	1.17	±	0.70	1.11	±	0.63	0.279
Sleep latency	0.73	±	0.76	0.74	±	0.76	0.64	±	0.76	0.66	±	0.74	0.69	±	0.72	0.382
Sleep duration	0.98	±	0.78	0.91	±	0.78	0.88	±	0.74	0.84	±	0.76	0.79	±	0.73	0.013
Habitual sleep efficiency	0.09	±	0.38	0.10	±	0.42	0.09	±	0.36	0.08	±	0.34	0.07	±	0.36	0.923
Sleep disturbances	0.93	±	0.51	0.94	±	0.50	0.88	±	0.51	0.91	±	0.50	0.87	±	0.50	0.204
Use of sleep medication	0.06	±	0.32	0.09	±	0.45	0.03	±	0.29	0.05	±	0.35	0.02	±	0.21	0.065
Daytime dysfunction	0.96	±	0.71	0.91	±	0.71	0.88	±	0.71	0.94	±	0.72	0.89	±	0.71	0.561
Dietary GL																
Sleep duration (hours)	6.3	±	0.8	6.3	±	0.8	6.3	±	0.8	6.4	±	0.8	6.4	±	0.8	0.067
PSQI-J																
Subjective sleep quality	1.15	±	0.66	1.19	±	0.66	1.13	±	0.62	1.15	±	0.64	1.20	±	0.71	0.515
Sleep latency	0.71	±	0.75	0.76	±	0.77	0.62	±	0.74	0.67	±	0.73	0.71	±	0.77	0.159
Sleep duration	0.94	±	0.81	0.91	±	0.74	0.87	±	0.72	0.82	±	0.76	0.85	±	0.77	0.241
Habitual sleep efficiency	0.12	±	0.46	0.09	±	0.39	0.07	±	0.34	0.09	±	0.35	0.07	±	0.30	0.370
Sleep disturbances	0.87	±	0.50	0.92	±	0.48	0.86	±	0.52	0.96	±	0.48	0.91	±	0.53	0.041
Use of sleep medication	0.04	±	0.33	0.07	±	0.37	0.05	±	0.32	0.06	±	0.40	0.02	±	0.20	0.388
Daytime dysfunction	0.94	±	0.72	0.88	±	0.70	0.89	±	0.71	0.92	±	0.71	0.96	±	0.73	0.537

PSQI-J, the Japanese version of the Pittsburgh Sleep Quality Index; GI, glycemic index; GL, glycemic load; Q: quintile.

a Linear regression analyses were used to assess the linear trends between sleep duration and each PSQI-J component score across the quintiles of starchy food intake, dietary GI, and dietary GL by using the median value of each quintile.

The multivariate-adjusted ORs (quintile, 95%CI) for the prevalence of poor sleep across the quintiles of rice intake were 1.00 (reference), 0.68 (0.49–0.93), 0.61 (0.43–0.85), 0.59 (0.42–0.85), and 0.54 (0.37–0.81), respectively, indicating that rice intake had a significant positive association with better sleep (*p* for linear trend = 0.015) (Table 3). In contrast, the multivariate-adjusted ORs across the quintiles of noodle intake were 1.00 (reference), 1.25 (0.90–1.74), 1.05 (0.75–1.47), 1.31 (0.94–1.82), and 1.82 (1.31–2.51), respectively, indicating that noodle consumption was significantly associated with poor sleep (*p* for linear trend = 0.002) (Table 3). These associations did not change even after further adjusting for vegetable, meat, and fish intake (data not shown). Dietary GI was also associated with good sleep (*p* for trend = 0.020), whereas dietary GL was not (*p* for trend = 0.092).

**Table 3:** ORs for the prevalence of poor sleep^a^ in each quintile of dietary rice, bread, and noodle intake as well as dietary glycemic index and glycemic load.

	Q1	Q2	Q3	Q4	Q5	*p* b value	Continuous^c^	*P* value
	(lowest)		(middle)		(highest)		(1 SD increment)	
Rice								
Prevalence of poor sleep (%)	39.0	30.7	28.0	28.0	26.0	<0.001		
Age-, sex-adjusted OR	1	0.69	0.60	0.59	0.54	0.001	0.85	0.010
(95%CI)	(reference)	(0.51, 0.94)	(0.44, 0.82)	(0.43, 0.81)	(0.39, 0.74)		(0.76, 0.96)	
Multivariate-adjusted OR^d^	1	0.68	0.61	0.59	0.54	0.015	0.87	0.045
(95%CI)	(reference)	(0.49, 0.93)	(0.43, 0.85)	(0.42, 0.85)	(0.37, 0.81)		(0.76, 1.00)	
Bread								
Prevalence of poor sleep (%)	27.7	30.1	29.5	31.3	33.3	0.284		
Age-, sex-adjusted OR	1	1.13	1.10	1.19	1.31	0.545	1.06	0.312
(95%CI)	(reference)	(0.82, 1.55)	(0.80, 1.51)	(0.87, 1.63)	(0.96, 1.78)		(0.95, 1.19)	
Multivariate-adjusted OR^d^	1	1.14	1.04	1.05	1.14	0.921	1.01	0.885
(95%CI)	(reference)	(0.81, 1.60)	(0.74, 1.47)	(0.74, 1.50)	(0.79, 1.63)		(0.89, 1.14)	
Noodle								
Prevalence of poor sleep (%)	24.6	30.5	26.4	31.3	39.0	<0.001		
Age-, sex-adjusted OR	1	1.35	1.10	1.38	1.95	<0.001	1.21	0.001
(95%CI)	(reference)	(0.97, 1.87)	(0.79, 1.53)	(1.00, 1.91)	(1.42, 2.67)		(1.09, 1.35)	
Multivariate-adjusted OR^d^	1	1.25	1.05	1.31	1.82	0.002	1.21	<0.001
95%CI)	(reference)	(0.90, 1.74)	(0.75, 1.47)	(0.94, 1.82)	(1.31, 2.51)		(1.09, 1.35)	
Dietary GI								
Prevalence of poor sleep (%)	35.4	34.3	24.7	28.9	28.5	0.016		
Age-, sex-adjusted OR	1	0.96	0.59	0.73	0.72	0.006	0.82	<0.001
(95%CI)	(reference)	(0.71, 1.30)	(0.43, 0.81)	(0.54, 1.00)	(0.52, 0.99)		(0.74, 0.91)	
Multivariate-adjusted OR^d^	1	0.96	0.60	0.77	0.77	0.020	0.85	0.006
(95%CI)	(reference)	(0.71, 1.31)	(0.44, 0.84)	(0.56, 1.07)	(0.55, 1.07)		(0.76, 0.95)	
Dietary GL								
Prevalence of poor sleep (%)	31.1	30.0	25.8	31.1	33.9	0.379		
Age-, sex-adjusted OR	1	0.96	0.78	1.02	1.14	0.215	1.02	0.721
(95%CI)	(reference)	(0.70, 1.32)	(0.56, 1.08)	(0.74, 1.40)	(0.83, 1.57)		(0.91, 1.14)	
Multivariate-adjusted OR^d^	1	0.98	0.79	1.09	1.27	0.092	1.07	0.284
(95%CI)	(reference)	(0.71, 1.35)	(0.56, 1.11)	(0.77, 1.54)	(0.88, 1.82)		(0.95, 1.21)	

GI, glycemic index; GL, glycemic load, CI, confidence interval; SD, standard deviation; OR: odds ratio; PSQI-J: Japanese version of the Pittsburgh Sleep Quality Index; Q: quintile;

a A PSQI-J global score >5.5 indicate poor sleep.

b The *χ* ^2^ test was used to analyze the prevalence of poor sleep, and logistic regression analysis was used to assess the linear trends of ORs by using the median value of each quintile.

c Differences of SD for rice, bread, noodles, dietary GI, and dietary GL were 69.1 g/1,000 kcal, 26.2 g/1,000 kcal, 32.8 g/1,000 kcal, 3.9, and 43.8/1,000 kcal, respectively.

d Multivariate models included age (continuous), sex (continuous), Body mass index (kg/m^2^; continuous), smoking status (i.e., current, previous, or never; dummy variable), habitual exercise (MET-h/week; continuous), alcohol consumption (percentage of energy; continuous), frequency of breakfast consumption (i.e., 0–3, 4–6, or 7 days/week; dummy variable), rice intake (for the multivariate analyses of bread and noodles; continuous), bread intake (for the multivariate analyses of rice and noodles; continuous), and noodle intake (for the multivariate analyses of rice and bread; continuous).

## Discussion

This study evaluated the association between sleep quality and the intake of common starchy foods (i.e., rice, bread, and noodles) as well as the dietary GI and GL in a Japanese population. Rice consumption was positively associated with sleep quality. In contrast, noodle consumption had a significant inverse association with sleep quality. Furthermore, a significant positive relationship between dietary GI and sleep quality was observed. Because rice is a major contributor to the dietary GI among Japanese people, differences between the GI values of rice, bread, and noodles may influence their sleep quality.

The present results show that higher consumption of rice, which is the main contributor to the dietary GI in Japanese foods, and the dietary GI itself are closely associated with a lower PSQI-J global score, i.e., good sleep. In a previous cross-sectional study of children younger than 2 years of age, the consumption of an evening meal with a high-GI was associated with longer sleep duration compared with the consumption of an evening meal with a low-GI [9]. The present results are consistent with these previous findings, because rice intake and the dietary GI were associated with sleep duration but not sleep latency in the present study. In contrast, in a clinical trial of 12 healthy young men, a carbohydrate-based meal with a high-GI was significantly associated with a shortening of sleep onset latency compared with a meal with a low-GI and was most effective when consumed 4 hours before going to sleep [10]. We assessed daily rice consumption and the dietary GI, but not rice consumption or the GI of evening meals, which may have affected the present results.

A high dietary GI may affect sleep quality via the effects of tryptophan (TRP) and melatonin [30]–[36]. A previous study showed that both carbohydrate intake and a meal with a high-GI increase the ratio of TRP to other large neutral amino acids (TRP/LNAA) after the meal, compared with a meal with a low-GI [37]. LNAA and TRP are competitively transported across the blood–brain barrier, and a higher TRP/LNAA ratio would result in more TRP being transported into the brain. In the brain, TRP is converted into serotonin and then to melatonin, which induces sleep [30]–[36].

Similar to the dietary GI, rice consumption was significantly associated with a low PSQI-J global score in the present study. Rice, especially white rice, is a common starchy food eaten by Japanese people; it accounts for approximately 28% of the daily energy intake and 70% of cereal intake [17]. Furthermore, white rice accounts for 59% of the dietary GI for in the Japanese diet [11]–[13]. Therefore, rice intake would affect sleep quality via the effect of the GI. In addition, rice contains high levels of melatonin [38], which may also favor good sleep.

In contrast, bread intake was not significantly associated with sleep quality, whereas noodle intake was significantly associated with poor sleep. The GIs of the breads and noodles used in the analyses ranged from 51 to 74 and 46 to 47, respectively; these values are lower than 77, which is the GI of Japanese white rice [11]. In a previous study, the TRP/LNAA ratio increased by 17% after a mixed-macronutrient meal with a high-GI (GI = 70) and was higher than that after a mixed-macronutrient meal with a low-GI (GI of 50; 8% increase), even though the amount of carbohydrates was the same in both meals (66.5% energy) [37]. Although noodles are major starchy foods, the GI of noodles is too low to increase the postprandial TRP/LNAA ratio. Furthermore, noodle intake was inversely associated with rice intake. In the present study, higher noodle intake was associated with poor sleep even after adjusting for rice intake. However, it is possible that the adjustment using statistical models is insufficient; thus, the low sleep quality of subjects with higher noodle intake may be due to a lower rice intake. An interventional study is required to investigate the differences in the associations of starchy foods with sleep quality.

Shorter sleep duration is associated with a relative increase and decrease in calories derived from fat and carbohydrates, respectively [39]. In Japan, breakfast often consists of foods low in fat but high in carbohydrates and fiber. Japanese people who eat breakfast generally consume more rice [40]. People with good sleep quality tend to eat breakfast, which may affect the association between rice intake and sleep quality. However, in the present study, lower rice intake was significantly associated with poor sleep even after adjusting for the frequency of breakfast consumption.

In this study, diets with high rice intake and a high GI were significantly associated with good sleep; however, such diets are also reported to be associated with several health problems including obesity, diabetes mellitus, cardiovascular disease, and some cancers [11]–[16], [41]–[43]. Furthermore, obesity induced by the long-term consumption of a diet with a high-GI may cause sleep apnea syndrome, which may also affect sleep quality. Accordingly, the association between the long-term consumption of meals with a high-GI and sleep quality should be analyzed in greater detail.

One of the strengths of the present study is that we examined rice consumption in a large Japanese population. Japanese people consume approximately 10 times more rice than European and North American people [44]. Thus, it is important to evaluate the association between rice and sleep in people with high rice consumption. Further, this is the first study to investigate the association between rice, bread, and noodle consumption and sleep quality. In addition, the GI and GL were calculated by using responses to a validated questionnaire [21]. Nevertheless, the present study also has several limitations. First, we restricted the final study population to white-collar workers; white-collar work is reported to be strongly correlated with poor sleep quality [45]. Compared with the general Japanese population, the study participants had a similar mean PSQI-J global score but shorter mean sleep duration [46]. Therefore, the results of this study cannot necessarily be generalized to the overall Japanese population. Second, sleep quality is reported to be related to physiological actions and eating behaviors such as skipping meals, eating speed, and watching television during meals [47]; we did not have data on these variables. Third, the DHQ and PSQI-J were evaluated approximately 1 year apart. However, lifestyle factors such as dietary habits and sleep quality are unlikely to change much in 1 year among steadily employed middle-aged people. Fourth, women are reported to have difficulty sleeping at the beginning and end of their menstrual cycle [48], [49]; in the present study, we did not obtain data on the menstrual cycles of the female participants. However, when we compared the PSQI-J component and global scores of men and women, nearly identical trends were observed (data not shown). Fifth, the dietary GI and rice consumption data used in this study were daily values, not evening values. As mentioned in the preceding text, the consumption of a meal with a high-GI within 4 hours of going to bed may be an effective way of facilitating sleep [10]. In the present study, the dietary GI and rice intake were significantly associated with sleep duration but not sleep latency. However, dietary intake at dinner may be more closely associated with sleep quality.

In conclusion, the present study indicate that high consumption of rice and a high dietary GI are associated with good sleep, especially good sleep duration. Meanwhile, higher noodle consumption is associated with poor sleep quality. The effects of starchy foods on sleep may differ according to their GI values. Diets with a high-GI, especially those with high rice intake, may contribute to good sleep. Nevertheless, further interventional studies are required to determine appropriate carbohydrate intake during the evening meal to facilitate good sleep.
